# Luciferase-Based Detection of Antibodies for the Diagnosis of HPV-Associated Head and Neck Squamous Cell Carcinoma

**DOI:** 10.3390/diagnostics9030089

**Published:** 2019-08-06

**Authors:** Peter D. Burbelo, Adrija Chaturvedi, Abner L. Notkins, Sreenivasulu Gunti

**Affiliations:** 1 Dental Clinical Research Core, National Institutes of Dental and Craniofacial Research, National Institutes of Health, Bethesda, MD 20892, USA; 2 Experimental Medicine Section, National Institutes of Dental and Craniofacial Research, National Institutes of Health, Bethesda, MD 20892, USA

**Keywords:** tumor antigens, head and neck cancer, human papilloma virus-16 (HPV-16), luciferase immunoprecipitation systems (LIPS)

## Abstract

Point-of-care tests are needed for the screening of head and neck squamous cell carcinoma (HNSCC) and other malignancies. Luciferase immunoprecipitation systems (LIPS), employing light-emitting proteins, were used to examine serum antibodies against several cancer-associated targets in blood donor controls and subjects with colon cancer (CC) and HNSCC. The assessment of antibodies against the wild type p53 tumor antigen showed that approximately 25% of the CC and 20% of the HNSCC patients were seropositive. In addition, humoral responses against two p53 mutants, p53-R175H and p53-R273H, generally tracked the antibody responses seen against wild type p53. Analysis of antibodies against highly specific biomarkers of HPV-16-associated malignancy, E2, E6, and E7 oncoproteins, revealed no seropositivity in blood donors and CC patients. However, 45% (9/20) of the HNSCC patients showed E6 seropositivity, which overlapped all the detectable E2 (40%; 8/20) and E7 seropositive subjects (35%; 7/20). Using neodymium magnets, ultrarapid LIPSTICKS testing of HPV-16 E6 antibodies in <60 s per HNSCC sample demonstrated almost the same diagnostic performance (40% sensitivity and 100% specificity) as LIPS testing in 2.5 h. While additional improvements and standardization are needed, these results highlight the possibility of using these approaches for the diagnosis of HPV-16-associated HNSCC.

## 1. Introduction

There is an interest in developing point-of-care tests in the oral health setting for the clinical detection of head and neck squamous cell carcinoma (HNSCC) [1]. Based on the unchanging survival rates of HNSCC, there is a need for better diagnostic and prognostic tools [2,3]. One potential approach exploits the patient’s antibody responses directed against tumor antigens. Cancer-related antibodies against these tumor antigens are thought to arise by protein overexpression, altered spliced forms, mutations, protein modifications and/or represent viral oncogenes [4]. Studies in HNSCC and other cancers have detected antibodies against p53, a well-characterized tumor antigen that is commonly mutated in cancer [5]. In addition, human papillomavirus (HPV), particularly the HPV-16 and HPV-18 genotypes, are directly involved in causing cancer in a subset of HNSCC patients [6,7,8], as well as in cervical cancer and anal cancer [9]. Particularly relevant is the finding that serum antibodies against the HPV-16 E6 and HPV-16 E7 oncoproteins are highly specific biomarkers for the presence of HPV-driven tumors. Antibodies against HPV-16 E6 and E7 proteins are only found in patients harboring HPV-associated cancers and are not found in subjects with HPV infection alone without malignancy and healthy controls [8,10,11]. Interestingly, HPV-16 E6 antibodies can be detected decades before the future diagnosis of HNSCC, highlighting the potentially important role as an early biomarker for cancer screening [12,13].

We and others have used a fluid phase immunoassay employing luciferase-tagged recombinant fusion proteins, called luciferase immunoprecipitation systems (LIPS), for diagnostic purposes [14]. Owing to the enhanced presentation of both linear and conformational antigenic epitopes in solution, the LIPS immunoassay often has higher sensitivity, specificity and dynamic range of detection than solid-phase immunoassays. The antibody profiles generated by LIPS have been highly informative in elucidating patient subsets in autoimmune and infectious diseases [15,16,17,18], the discovery of new autoimmune conditions [19], the identification of novel pathogens [20,21], and for the diagnosis of pandemic viruses [22,23]. Recently, a streamlined version, LIPSTICKS, employing magnetic capture of immune complexes, was able to quickly and accurately measure diagnostic antibodies associated with six different autoimmune and infectious diseases, including for the diagnosis of Sjögren’s syndrome [24]. In this report, we describe studies using the LIPS assay to detect cancer-associated antibody responses against the tumor antigens including p53 and HPV-16 E2, E6 and E7 proteins. Additionally, the detection of HPV E6 antibodies by the LIPSTICKS technology enabled the rapid diagnosis of HPV-associated HNSCC.

## 2. Materials and Methods

### 2.1. Ethics Statement and Human Serum Samples

Informed written consent was obtained from all subjects in accordance with the human experimentation guidelines of the Department of Health and Human Services under IRB-approved protocols, and the studies were conducted according to the principles expressed in the Declaration of Helsinki. All human samples utilized in this report represented de-identified serum specimens with annotated data. Healthy volunteer (HV) blood donor controls were obtained from the NIH Blood Bank, Clinical Center, NIH, Bethesda, Maryland under NCT00001846 (entitled “Collection and distribution of blood components from healthy donors for in vitro research use”). The serum samples from colon cancer (CC), systemic lupus erythematosus (SLE), and HNSCC patients used in this report were obtained from Conversant Bio (601 Genome Way Suite 1200, Huntsville, Alabama 35806). Subjects provided written informed consent under the Western Institutional Review Board approved by Conversant Bio. All human serum samples from Conversant Bio were collected, processed, and distributed in full ethical and regulatory compliance with the sites from which they were collected. This includes independent ethical review, Institutional Review Board approval (where appropriate), independent regulatory review, and Conversant Bioethical review.

The HV controls (*n* = 20) from the NIH Blood bank represented subjects with an average age of 57 years and were 80% male. For the subjects with HNSCC (*n* = 20), the average age of cancer diagnosis was 61 years and 75% were male. The clinical information on the CC (*n* = 20) was not available. The serum samples from SLE patients (*n* = 20) had an average age of 47.1 years and were 100% female.

### 2.2. DNA Constructs for Luciferase-Tagged Antigens

Previously described pREN2 constructs for *Renilla* luciferase (*Ruc*) and p53 chimeric fusion protein were used [25]. Two known p53 cancer mutant proteins, p53-R175H and p53-R273H, were also generated in pREN2. The coding sequences for E2, E6 and E7 proteins from HPV-16 were subcloned into the pREN2 vector for generating C-terminal HPV fusion proteins essentially as described for other antigens [25]. The E6 cDNA was also subcloned into the pNano2 vector for expression as a NanoLuc fusion protein [24], a highly active luciferase. The integrity of the described constructs was confirmed by DNA sequencing.

### 2.3. LIPS Analysis

Essentially as described, the LIPS assays were performed in approximately 2.5 h using a 96-well plate format at room temperature [19,20,21]. Briefly, recombinant luciferase antigen lysates were produced from transfection of DNA plasmids constructs into Cos1 cells and their activity in light units (LU) was determined with a tube luminometer (Turner Design 20/20). To initiate testing, 40 μL of buffer A, 10 μL of diluted human sera (1 μL equivalent), and the luciferase-antigen cell extract (input of approximately 10 million LU), diluted in buffer A, were added to each well of a microtiter plate for 1 h. Next, a 30% suspension (6 μL) of Ultralink protein A/G beads (Pierce Biotechnology, Rockford, IL, USA) was added to the bottom of each well of a 96-well filter HTS plate (Millipore, Bedford, MA, USA). The 100 μL antigen-antibody reaction mixture was moved to a filter plate and incubated for 1 h on a rotary shaker. Multiple washing steps of the retained protein A/G beads were then performed. After completion of the washing steps, LU were measured in a Berthold LB 960 Centro microplate luminometer (Berthold Technologies, Bad Wildbad, Germany) using either coelenterazine or furimazine substrate (Promega, Madison, WI, USA) for detecting Renilla luciferase and NanoLuc activity, respectively.

### 2.4. Detection of HPV E6 Antibodies by LIPSTICKS

The LIPSTICKS technology [24] employed cell extract containing NanoLuc-E6 protein was investigated for ultrarapid diagnosis of HPV-associated HNSCC. Compared to the previously reported protocol, a slightly modified version of the assay was used which changed the order of reagent addition and produced a higher signal. To perform the modified version of the assay, 5 μL of the diluted serum sample (1:50 in water) is added to 5 μL of the NanoLuc-E6 fusion protein cell extract (30 million LU/ μL) in a 1.5 mL microfuge tube and then 5 μL of diluted paramagnetic beads (Thermo Scientific/Pierce^®^ protein A/G magnetic beads, Waltham, MA, USA), diluted 1:5 in water are added. The reaction mix is tapped two times to disperse the magnetic beads and then 100 μL of buffer A is pipetted into the reaction mixture and the tube is immediately vortexed for 2 s. A 1/8 diameter neodymium magnetic stick (K & G Magnets, Pipersville, PA, USA) is then immersed into the tube containing the beads for 5 s to collect the immune complexes. The magnet is removed and dipped twice in wash buffer A. Lastly, the magnetic stick is placed in a tube, preloaded with 100 μL of the Nanoglow substrate (Promega) and the luminescent glow is measured with the tube luminometer with an integration time of 1 s.

### 2.5. Data Analysis

GraphPad Prism 6 software (San Diego, CA, USA) was employed for data plotting and statistical analysis. Non-parametric Mann-Whitney *U* tests were used to compare the antibody levels among groups and only statistically significant (*p* < 0.05) are shown in the figures. For calculations of sensitivity and specificity, cut-off limits for each antigen were derived from the mean value plus three standard deviations of the healthy blood donor controls [26].

## 3. Results

**Figure 1:**
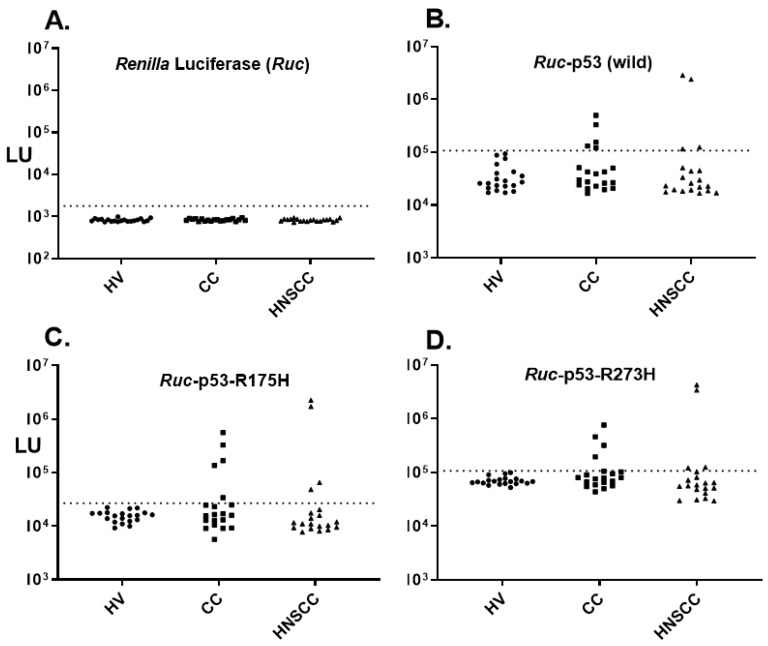
Antibodies against wild type p53 and mutant p53 proteins. Antibodies were analyzed by luciferase immunoprecipitation systems (LIPS) against (**A**) *Renilla* luciferase, (**B**) wild type p53, (**C**) p53-R175H, and (**D**) p53-R273H. Each symbol represents individual samples from twenty HV controls, twenty colon cancer (CC) patients and twenty head and neck squamous cell carcinoma (HNSCC) patients. Antibody levels in light units (LU) are plotted on the Y-axis using a log_10_ scale. The cut-off value for each antigen is shown by the dotted line and was based on the mean plus three standard deviations of the blood donor controls.

Using the LIPS technology, antibodies against wild type p53 were evaluated in serum samples from a cohort that included HV (*n* = 20), and CC (*n* = 20) and HNSCC (*n* = 20). Testing an extract containing only *Renilla* luciferase (*Ruc*) as a control protein revealed low levels of antibodies in the HV, CC and HNSCC subjects (Figure 1A). LIPS analysis of the *Ruc*-p53 protein target demonstrated relatively low antibody levels in HV, whereas a small number of the patients with CC and HNSCC had much higher antibody levels (Figure 1B). To determine the seropositivity of p53 antibodies in the cancer subjects, a cut-off value was assigned based on the antibody values corresponding to the mean plus three standard deviations of the 20 HV controls. As shown, 25% (5/20) of the CC and 20% (4/20) of HNSCC patients were p53 seropositive with a diagnostic specificity of 100% (Figure 1B). Three subjects with CC and two with HNSCC showed p53 seropositive autoantibodies that were just above the cut-off value. To determine whether the antibody signal detecting p53 antibodies might be enhanced, two cancer mutant variants of p53, p53-R175H, and p53-R273H, were tested (Figure 1C,D). However as shown, the antibody profile against p53-R175H and p53-R273H, for the most part, showed a similar profile as wild type p53 protein.

**Figure 2:**
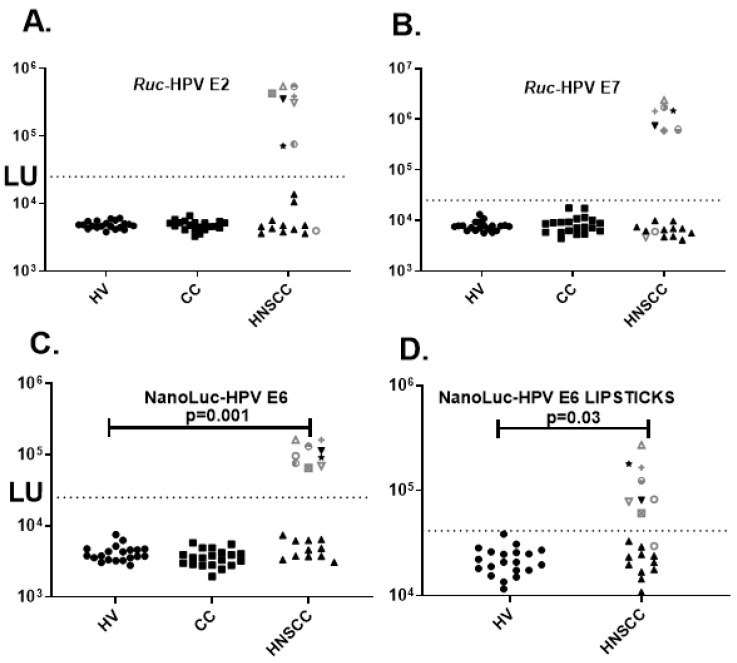
LIPS and LIPSTICKS detection of antibodies against HPV-16 oncoproteins. Antibodies were analyzed by LIPS in the HV, CC and HNSCC patients against (**A**) HPV E2, (**B**) HPV E7, (**C**) HPV-E6. (**D**) NanoLuc-HPV E6 LIPSTICKS testing of the HV and HNSCC. Antibody levels in LU are plotted on the log_10_ scale Y-axis. The cut-off values are shown by the dotted line, which were based on the mean plus three standard deviations of the blood donor controls. For comparison amongst the 4 different tests, the nine HPV seropositive detected by the HPV E6 LIPS test shown in panel C are shown by unique symbols.

Due to the known high prevalence of HPV-associated HNSCC, antibodies against E2, E7, and E6 HPV-16 proteins were analyzed. LIPS analysis of HPV-16 E2 antibodies revealed low levels in the HV and CC patients that were similar to the buffer blanks (Figure 2A and data not shown). However, eight (45%) of the HNSCC patients showed high E2 antibody levels that were approximately 20–100 times higher than the corresponding mean levels of the HV controls (Figure 2A). LIPS analysis of antibodies against the second HPV-16 antigen, E7, also did not detect seropositives in the HV control or CC patients but detected seven (35%) seropositive HNSCC patients which overlapped the E2 seropositive subjects (Figure 2B). Testing of the E6 HPV antigen as a *Renilla* luciferase fusion protein revealed the same pattern with HV and CC showing seronegativity yet detected nine (45%) of the HNSCC as seropositive (data not shown). To further confirm this result, a different reporter, NanoLuc was employed to detect the HPV-16 E6 antibodies. As shown in Figure 2C, the NanoLuc-E6 test also revealed that nine (45%) HNSCC subjects that were seropositive. Testing of twenty SLE patients as another set of disease controls detected no E6 or E7 seropositivity further supporting the observation that the HPV-16 antibodies responses were associated with the HNSCC patients (data not shown). Inspection of the HPV antibody profile in the HNSCC subjects revealed that nine subjects were seropositive for E6, eight overlapping subjects were E2 seropositive and 7 subjects were seropositive for E7 protein. Lastly, the presence of nine E6 seropositive samples in the HNSCC group (45%) was statistically different than the complete absence of E6 seropositivity in either the HV group or subjects with colon cancer (Fischer’s Exact test, *p* = 0.002).

Based on the substantial difference in antibody levels seen by LIPS between the HPV-negative and HPV-positive HNSCC samples, we sought to determine whether the rapid LIPSTICKS format, employing the NanoLuc-E6 fusion protein, could be used for detection of these antibodies in the HNSCC group (Figure 2D). The one-minute LIPSTICKS assays generated antibody signals that differentiated the HV and HPV-negative HNSCC patients from the HPV-positive HNSCC samples, in which the mean level of anti-E6 antibodies in the HNSCC samples was significantly higher (*p* = 0.03) than the value seen in the twenty HV controls (Figure 2D). Using a cutoff value based on the mean plus three standard deviations of the HV controls revealed a diagnostic performance of 89% (8/9) sensitivity and 100% specificity for detecting the E6 antibody-positive HNSCC patients in the rapid format. Moreover, the E6 antibody levels detected by the LIPSTICKS format were significantly different (*p* < 0.0001) between the HPV-negative HNSCC and HPV-positive HNSCC cases (data not shown). These findings suggest that the E6 LIPSTICK test has the potential to diagnose HPV-16-associated HNSCC.

## 4. Discussion

Using the LIPS technology, antibody-based biomarkers against the human p53 tumor antigen and antibodies against the E2, E6 and E7 oncogenes of HPV were investigated. Approximately 25% of the colon cancer and 20% of the HNSCC patients harbored statistically significant autoantibodies against wild type p53 and is consistent with other published studies [27,28]. LIPS profiling against HPV E2, E7 and E6 viral proteins detected a high prevalence of antibody responses in HNSCC, but not in the HV, CC or SLE and is in agreement with other studies [8,12,29]. Consistent with published reports [12,13,30], HPV16 E6 antigen was found to have a higher sensitivity than the E2 and E7 antigen. The finding that 45% of the HNSCC subjects had HPV-16 associated cancer approximates matches the known prevalence of about 25% [31] and is likely due to the stochastic nature of our small cohort. Particularly encouraging was the quite robust detection of HPV-16 antibodies by LIPS in the HNSCC subjects highlighting the potential ability of the assay to screen for these antibodies in a high-throughput fashion.

There is also great interest in rapid point of care tests for infection, cancer, and autoimmunity [32]. The most common POC directed at diagnostic antibodies are based on lateral flow and microfluidic devices. One recent study showed that a microfluidic device coupled with immunoassay detected HPV-16 E6 antibodies with moderate diagnostic sensitivity and specificity but required at least an hour for processing [10]. Here, the one-minute LIPSTICKS assay performed quite well to detect HPV-16 E6 antibodies associated with HNSCC. While some of the HNSCC subjects showed relatively dramatic differences between the negative and positive samples, other subjects were less easily detected as seropositive and fell near the cut-off value and one sample was negative. We anticipate that further improvements in the E6 HPV-16 LIPSTICK assay related to antigen design and assay standardization will further enhance test performance. One limitation of our study was the relatively small cohort that was tested. Exploring whether LIPSTICKS can detect HPV-associated HNSCC in a larger well-characterized cohort is warranted.
